# Validation study of “Santé-Cerveau”, a digital tool for early cognitive changes identification

**DOI:** 10.1186/s13195-023-01204-x

**Published:** 2023-04-03

**Authors:** Constance Lesoil, Stéphanie Bombois, Octave Guinebretiere, Marion Houot, Mahsa Bahrami, Marcel Levy, Rémy Genthon, Frédérique Bozon, Heidy Jean-Marie, Stéphane Epelbaum, Pierre Foulon, Nicolas Villain, Bruno Dubois

**Affiliations:** 1 Assistance Publique-Hôpitaux de Paris (AP-HP), Department of Neurology, Institute of Memory and Alzheimer’s Disease (IM2A), Paris, France; 2 INSERM U1171 - Degenerative and Vascular Cognitive Disorders, University of Lille, Lille, France; 3 Centre of Excellence of Neurodegenerative Disease (CoEN), AP-HP, Pitié-Salpêtrière, Institut du Cerveau - Paris Brain Institute - ICM, Paris, France; 4 MindMaze France, Paris, France; 5 Sorbonne Université, INSERM U1127, CNRS 7225, Paris, France

**Keywords:** Alzheimer’s disease, Early diagnosis, Digital tool, Cognitive complaint, Mild dementia, Memory, COVID-19, Care plan

## Abstract

**Background:**

There is a need for a reliable, easy-to-use, widely available, and validated tool for timely cognitive impairment identification. We created a computerized cognitive screening tool (Santé-Cerveau digital tool (SCD-T)) including validated questionnaires and the following neuropsychological tests: 5 Word Test (5-WT) for episodic memory, Trail Making Test (TMT) for executive functions, and a number coding test (NCT) adapted from the Digit Symbol Substitution Test for global intellectual efficiency. This study aimed to evaluate the performance of SCD-T to identify cognitive deficit and to determine its usability.

**Methods:**

Three groups were constituted including 65 elderly Controls, 64 patients with neurodegenerative diseases (NDG): 50 AD and 14 non-AD, and 20 post-COVID-19 patients. The minimum MMSE score for inclusion was 20. Association between computerized SCD-T cognitive tests and their standard equivalent was assessed using Pearson's correlation coefficients. Two algorithms (a simple clinician-guided algorithm involving the 5-WT and the NCT; and a machine learning classifier based on 8 scores from the SCD-T tests extracted from a multiple logistic regression model, and data from the SCD-T questionnaires) were evaluated. The acceptability of SCD-T was investigated through a questionnaire and scale.

**Results:**

AD and non-AD participants were older (mean ± standard deviation (SD): 72.61 ± 6.79 vs 69.91 ± 4.86 years old, *p* = 0.011) and had a lower MMSE score (Mean difference estimate ± standard error: 1.74 ± 0.14, *p* < 0.001) than Controls; post-COVID-19 patients were younger than Controls (mean ± SD: 45.07 ± 11.36 years old, *p* < 0.001). All the computerized SCD-T cognitive tests were significantly associated with their reference version. In the pooled Controls and NDG group, the correlation coefficient was 0.84 for verbal memory, -0.60 for executive functions, and 0.72 for global intellectual efficiency. The clinician-guided algorithm demonstrated 94.4% ± 3.8% sensitivity and 80.5% ± 8.7% specificity, and the machine learning classifier 96.8% ± 3.9% sensitivity and 90.7% ± 5.8% specificity. The acceptability of SCD-T was good to excellent.

**Conclusions:**

We demonstrate the high accuracy of SCD-T in screening cognitive disorders and its good acceptance even in individuals with prodromal and mild dementia stages. SCD-T would be useful in primary care to faster refer subjects with significant cognitive impairment (and limit unnecessary referrals) to specialized consultation, improve the AD care pathway and the pre-screening in clinical trials.

## Background

Alzheimer's disease (AD) affects nearly one million people in France and represents a major public health issue. Less than half of the patients are diagnosed in France with a mean Mini Mental State Examination (MMSE) score of 17 at the time of diagnosis [1], i.e. at a moderately advanced stage of disease. Early diagnosis of cognitive and memory impairment is an important health concern due to the impact on patients, caregivers, and healthcare systems [2, 3].

Early detection is essential to set up a care plan, prevent risky behaviors, and anticipate complications arising from neurodegenerative diseases. Moreover, from a therapeutic point of view, preventive measures are already applicable in the life course [4], and AD disease-modifiers molecules are developed for the earliest stages of the disease (MMSE > 20). For instance, the current anti-amyloid immunotherapies phase 3 pre-registration randomized-controlled clinical trials closest to approval involve only individuals with prodromal AD and mild AD dementia [5]. If these drugs were to be approved in the upcoming years, the need for wider access for the population to an early-stage biomarker-proven AD diagnosis would be huge [6].

However, the need for an early-stage diagnosis of AD comes up against several difficulties concerning the mobilization of patients, their families, and general practitioners who often need to be convinced of its value. General practitioners do not always have the time, training, or tools to do this [7, 8], notably because it may be difficult to distinguish between subjective memory complaints and objective memory deficits in the absence of formal memory testing [9]. Cognitive complaints are widespread in the elderly population; however, cognitive disorders are under-diagnosed [10], primarily due to the lack of cognitive assessment in primary care or to the use of scales inaccurate at detecting early-stage dementia [8]. To rebalance these two observations, it would be useful for any person with a cognitive complaint to have access to an objective and reliable evaluation in primary care. Brief computerized cognitive testing may be an option, and many tools are available today. However, most of them still need to be validated in large, controlled study settings, to allow their widespread use [11, 12].

We have developed, in partnership with MindMaze France, the "Santé-Cerveau" digital tool (SCD-T), including questionnaires and three cognitive tests adapted from validated paper/pencil versions: the 5 Word Test (5-WT), the Trail Making Test (TMT), and the Number Coding Test (NCT) adapted from the Digit Symbol Substitution Test (DSST), selected for their ability to assess significant cognitive functions e.g., episodic memory, executive functions and general intellectual efficiency, respectively. Their dysfunction is a proven early marker of AD and related disorders.

The objectives of the study were to evaluate 1) the SCD-T concordance with standard neuropsychological testing, 2) its performance to identify significant cognitive impairment in three different groups of individuals: controls, patients with neurodegenerative diseases, and subjects with a cognitive complaint after SARS-CoV-2 infection. Many subjects reported cognitive complaint after COVID-19 recovery, which was shown to be associated with affective symptoms (anxiety, depression, fatigue) or with impairment in a wide range of cognitive domains (executive functions, speed of processing, attention, memory, and processing abilities). The cognitive symptoms do not seem to be part of a neurodegenerative process, but could be related to functional, grey and white matter changes following axonal damage, inflammation or reduced perfusion [13]. Therefore, the pandemic occurrence provides an opportunity for us to evaluate the performance of our digital tool for discriminating memory complaint related to affective or attentional/executive disorders from memory complaints related to a true amnestic syndrome of AD. 3) its acceptability by users.

## Populations

To study the capacity of SCD-T to identify significant cognitive impairment and to discriminate changes associated with Alzheimer disease, we included in the study: i) subjects with cognitive deficits previously established by a comprehensive neuropsychological battery (CNB) and with a defined diagnosis based on our clinical work-up, i.e., patients with Alzheimer disease and non-Alzheimer neurodegenerative diseases at early clinical stages; and 2) ii) control subjects with normal cognitive functioning, with or without memory complaint. In addition, as mentioned above, the occurrence of the pandemic COVID-19 infection was an opportunity to include a third group of subjects with SARS CoV2 infection. All the participants (*n* = 149) in the SCD-T validation study were consecutively recruited in the context of clinical routine at the IM2A between February 2020 and April 2021.

To be included in the study, all participants had to be between 60 and 85 years of age (excepted for post-COVID-19 patients who had no age limits), registered to the French National Health Insurance system, signed the written consent form, be a native French speaker, with > 7 years of education, and have a MMSE score ≥ 20 points. Patients with a known neurological condition other than AD or related diseases, history of neoplasia or cerebral radiotherapy, developmental disorders or severe psychiatric illness (including severe depressive syndromes), history of head trauma or stroke with sequelae were excluded. We also excluded the subjects with addiction (alcohol or drugs), visual or auditory sensory deficits that could prevent the performance of cognitive tests, and subjects taking medication at doses known to interfere with memory and concentration.

All the participants of this study were evaluated with a comprehensive neuropsychological battery (CNB). In case of an impaired cognitive performance, the clinical routine diagnostic work-up was completed with a psychological interview, a ^18^F-FDG PET-MRI, and a lumbar puncture for cerebrospinal fluid core AD biomarkers investigation (Aβ_1-42_ and total and phosphorylated tau proteins). The lumbar punctures were performed and analyzed based on a method described elsewhere [14]. The diagnosis was established after an interdisciplinary discussion according to international criteria [15–20].

### *Patients with neurodegenerative disease (NDG) (n= 64)*

The group included 50 patients with AD according to the International Working Group 2021 criteria [15] and confirmed by positive CSF biomarkers (25 patients at a prodromal stage, 25 patients at a mild dementia stage (MMSE between 20 and 26)), 14 patients with a related degenerative disease with normal CSF biomarkers, including 6 patients with Lewy body dementia [16], 3 patients with primary primary progressive aphasia [17], 2 patients with frontotemporal dementia [18], 2 patients with non-AD amnestic syndrome and 1 patient with cortico-basal degeneration [19].

### *Controls (n= 65)*

The group consisted of cognitively unimpaired individuals (normal performance on the CNB): 31 subjects with no memory complaints and 34 subjects with memory complaint and a CSF AD biomarkers investigation (32 within the normal range and 2 with abnormal levels (corresponding to individuals ‘asymptomatic at risk’ for AD, or preclinical AD).

### *Post-COVID-19 patients (n= 20)*

During the COVID-19 pandemic, we included 20 individuals with persistent cognitive complaints after 3 to 6 months post-SARS-CoV-2 infection, classified as post-COVID-19 condition [20]. We enrolled all the individuals with a SARS-CoV-2 infection confirmed by an RT-PCR test referred to IM2A for cognitive testing. The inclusion period was between November 2020 and April 2021.

## Methods

### "Santé-Cerveau" digital tool (SCD-T)

#### Settings

SCD-T is a CE marking, class I digital medical device. In this validation study, participants were presented with the prototype version of this device developed with MindMaze France. This tool is accessible from a web platform and is performed on a touch tablet (Android operating system; Samsung® Galaxy Tab S5e®; 10.5-inch screen) equipped with a standard-size headset (brand name) with an integrated microphone. Data recorded on SCD-T (test results, questionnaire responses) were transferred to the Curapy platform (www.Curapy.com) and available to the physician in the form of individual, automatically generated reports.

This platform, developed by MindMaze France, allows for data security (encryption, logging, secure operation) and uses an approved health data server (AZNetwork).

#### Contents

SCD-T includes questionnaires and cognitive tests to assess the intensity of the memory complaint, comorbid conditions, and the detection of objective memory and cognitive impairment.

##### *The questionnaires*

They include the participants’ socio-demographic (age, sex, level of education) and basic medical (personal medical history, family history of neurodegenerative disorder, and current treatments) data, the intensity of the memory complaint assessed with the Mac Nair 15-item scale [21], and the mood status measured with the 15-item Geriatric Depression Scale (GDS) [22]. Thus, the questionnaires consider factors associated with cognitive impairment, dementia and Alzheimer disease. Among them, some factors are modifiable and as such of particular interest to treat, as hypertension, diabetes, cardiovascular diseases and depression [23].

##### *The neuropsychological tests*

We selected three cognitive tests for their ability to assess the main cognitive functions known to be altered at the early stage of AD and related disorders (global intellectual efficiency, episodic memory, and executive functions). These validated paper/pencil neuropsychological tests were adapted and integrated into a digital version as close as possible to their original version, in terms of presentation, time spent on the test, and content of the instructions. Each of the evaluation tests was preceded by a presentation of the instructions with a video example, followed by a short training phase.

The number coding test (NCT) adapted from the Digit Symbol Substitution Test (DSST) [24] was proposed to test the overall intellectual efficiency through the speed of central processing and execution, visuospatial, and working memory functions. The DSST is presented as the most accurate predictor of brain dysfunction among the other Wechsler Adult Intelligence Scale (WAIS) subtests and is therefore considered a good tool to identify cognitive deficits in the older adult population [25]. Moreover, it has been one of the tests used to detect early cognitive changes associated with the progression from preclinical to prodromal stage of AD; it is also sensitive to show cognitive decline in prodromal and mild dementia [26]. In the digital version, a series of numbers were presented on the screen; the individual was instructed to associate each number with a symbol by selecting it from a list, using the code provided at the top of the screen. The number of total, good and wrong answers over a 2-min test was recorded.

The trail making test (TMT) [27] was proposed to assess executive functions known to be early impaired in AD [28] and in related disorders, such as frontotemporal dementias, cortico-basal and progressive supranuclear palsy syndromes [29]. In the digital version, the participant clicked on the numbers on the screen as quickly as possible following an ascending order (TMT-A), then alternated from the series of numbers following an ascending order, to the series of letters following an alphabetical order (TMT-B). We recorded the execution time (in seconds) of each parts A and B of the test and the calculated time of part B minus part A.

The 5 Word Test (5-WT) [21] is based on the principle of semantic cueing to identify an amnestic syndrome of the hippocampal type (ASHT) [30, 31]. ASHT was shown to be highly associated with AD pathology [32]. In the digital version, a list of five words was presented that the user has to read aloud and to name in response to their categories. An immediate recall of the five words, using an automated voice recognition system, controls for the correct encoding of the five words. The delayed recall of the list, with automated voice recognition, occurs after five minutes, corresponding to the NCT and TMT period. Semantic cues are used in the test phase to prompt recall of items not retrieved by free recall (cued recall). The scores include a raw total score (total words recalled: cued and free recall, in the immediate and delayed recall phases), and a weighted total score (total cued recalls + 2 × total free recalls).

SCD-T cognitive tests were performed in the following order: the 5-WT immediate recall (encoding phase), the TMT parts A and B, the NCT, and 5-WTdelayed recall (retrieval phase).

#### Cognitive test conditions

SCD-T was performed in a quiet environment, in the presence of an investigator who intervened only at the beginning and end of the session to launch and close the app. The participant filled in the different questionnaires and performed the cognitive tests proposed alone.

### Standard comprehensive neuropsychological battery (CNB)

All participants underwent a reference CNB that differed between the groups of participants according to their clinical status.

#### CNB for patients with neurodegenerative diseases (NDG)

The tests and questionnaires were part of the standard diagnostic and follow-up procedures of the Pitié-Salpêtrière Memory Clinic (Institut de la Mémoire et de la Maladie d’Alzheimer*-* IM2A). The cognitive testing included: the Mini Mental State Examination (MMSE) [33], the Digit and Visuo-spatial Spans [34], the 40-items semantic battery (BECS-GRECO) [35], the Free and Cued Selective Reminding Test (FCSRT) [36], a praxis assessment [37], the Frontal Assessment Battery (FAB) [38], the TMT part A and B [39], the Rey Complex Figure [40], a verbal fluency assessment [41]. The behavioral testing included: the Hospital Anxiety and Depression (HAD) scale [42], the Starkstein Apathy scale [43]. The functional testing included: the Instrumental Activities of Daily Living (IADL) [44] and the Amsterdam Instrumental Activity of Daily Living Questionnaire [45]. SCD-T was performed within four months of the CNB.

#### CNB for Control group

A reduced CNB was proposed. It consisted of six cognitive tests: the MMSE [33], the FCSRT [36], the subtest code of the WAIS-IV [46], the Paced Auditory Serial Addition Test (PASAT) [47], the FAB [38], and the Stroop Test [48]. This cognitive testing was performed within four months of SCD-T, to avoid the retest effect and interference with memory testing.

#### CNB for post-COVID-19 patients

We adapted the CNB in line with the first cognitive and emotional reports from COVID-19 patients [49]. The cognitive testing included: the MMSE [33], the Digit and Visuospatial Spans [34], the FCSRT [36], the Delayed Matching to Sample Task 48 [50], the PASAT [47], the DSST [24], the FAB [38], the TMT parts A and B [27], the Stroop test [48], a verbal fluency assessment [41], and the Facial Action Coding System [51]. The emotional and behavioral testing included: the Posttraumatic Stress disorder Checklist Scale [52], the Chalder fatigue scale [53], the HAD scale [42], and the French apathy Dimensional Scale [54]. SCD-T was performed within one month of the CNB.

### SCD-T acceptability

All the participants completed an unpublished Questionnaire on Cognitive Tests (QCT) to test the acceptability of SCD-T. The QCT included 5 questions: (1) *How do you think you did on these cognitive tests compared to others of your age? *(2)* Do you think tests’ results represent your memory and attention? *(3)* How did you feel during the tests? *(4)* Were the instructions clear? *(5)* Would it have been helpful if someone had explained the tests to you and answered your questions before you took them?* The participants answered these questions by choosing an answer among 5 options. They also completed the System Usability Scale (SUS) [55] to test the usability of SCD-T. This scale includes 10 questions with 1 to 5 Likert-scale answers, from strongly disagree [1] to strongly agree [5]. The SUS score varies from 0 to 100 and is considered as "excellent" if equal or higher to 86, good if ≥ 73, acceptable if ≥ 52 [55].

### Statistical analyses

Demographic and clinical characteristics were compared using Welch's t test for quantitative measures and Fisher's exact test for categorical measures, regarding Controls and NDG groups. To compare cognitive tests from SCD-T, those from CNB, as well as acceptability measures between both groups, we performed generalized linear models with age, gender, and education level (with three levels; level 1: ≤ 12 years of education, under the high school diploma; level 2: 13 to 17 years of education, between high school diploma and Master’s degree; level 3: > 17 years of education, higher than Master’s degree); and clinical group as independent variables and each measure as the dependent variable. To correct for multiple testing, the Benjamini–Hochberg procedure was applied. Besides, comparisons between Controls and post-COVID-19 group were performed. To account for the age discrepancy between these two groups (mean ± SD, Controls: 69.9 ± 4.9 vs post-COVID-19: 45.1 ± 11.4, *p* < 0.001), we transformed the raw scores into standardized scores from the reference CNB using validated norms, controlling either by age, education level, or both. Since, norms for tests with SCD-T execution do not exist, we used those from the reference CNB execution. Due to missing norms, especially for the middle-aged population, several standardized test scores could not be computed (DSST bad and total answers). To compare the standardized scores of the groups, we used the Mann–Whitney U test and corrected for multiple testing using the Benjamini–Hochberg procedure. SCD-T scores were compared to their equivalent in the reference CNB using Pearson's correlation coefficients in a pooled Controls and NDG group and in post-COVID-19 subjects. Correlations were performed between (i) NCT good answers and MMSE; (ii) TMT B-A time and FAB; (iii) total 5-WT score and FCSRT total recall. The Benjamini–Hochberg method was used to correct the p-values for statistical test multiplicity. Same approach was performed to compare NDG and post-COVID-19 groups on 5-WT.

The performances of SCD-T to discriminate NDG from Controls were studied through the development of two algorithms: one guided by clinicians and another one without a priori (i.e., classical machine learning classifier). The clinician-guided algorithm is intended to provide a reliable estimate of the clinical signature of AD. We first used the previously established 5-WT clinical threshold of 9 to identify an amnesic syndrome of hippocampal type. Second, we aimed to identify individuals with a dysexecutive syndrome (using NCT and TMT scores) among individuals with a score of 10 at the 5-WT. The machine learning algorithm was tested using a multiple logistic regression model, including the 8 scores of the 5-WT, TMT, and NCT cognitive tests and accounted for age, gender, education level, and medical comorbidities associated with AD (hypertension, diabetes, cardiovascular diseases, and depression). Contrary to the clinician-algorithm that relies on the domain-expert’s knowledge, the machine learning classifier used different kinds of variables (continuous cognitive tests, continuous, dichotomous, and ordinal socio-demographic variables, and dichotomous comorbidities) to extract knowledge and train the algorithm. We used fivefold cross-validation to optimized the threshold and test the algorithms’ performance. For the clinician-guided algorithm, threshold optimization was performed on NCT or TMT scores for subjects with a 5-WT equal to 10 on the training set. The machine learning algorithm threshold optimization was performed on the estimated probabilities extracted from the multiple logistic regression model on the training set. For both algorithms, the threshold optimization was performed to maximize specificity for a sensitivity of at least 95%. We set this level of sensitivity to avoid false negatives, i.e. falsely reassuring someone who should be consulting, while maximizing the specificity to maintain a low number of false positives. During training, the threshold is optimized in individuals from the training set (104 individuals), and then tested on subjects previously unseen by the model (test set: 25 individuals). This approach mimics the clinical situation, where the CNB is used to diagnose a cognitive impairment in a new patient. Performance indicators as sensitivity, specificity, positive predictive value (PPV) and negative predictive value (NPV) were assessed through the means and standard deviations of the fivefold cross-validation.

Statistical analyses were performed using R 3.6.1. (R Foundation for Statistical Computing, Vienna, Austria. URL https://www.R-project.org/.)

## Results

### Group comparisons

Table 1 shows the main characteristics of the study participants. The mean age of the participants was 71.3 years, with patients in the NDG group being significantly older compared to controls (mean ± SD: 72.6 ± 6.8 vs. 69.9 ± 4.9, respectively, *p* = 0.011). More men were included in the NDG group (54.7% vs. 32.3%, *p* = 0.013). There was no significant difference in education level between the groups of participants.

**Table 1:** Comparison between the Controls and the NDG groups on demographics, reference CNB, SCD-T and acceptability

	Controls *N* = 65 (50.39%)	NDG *N* = 64 (49.61%)	Estimate of the difference ± SE^∫^	p^‡^
**Demographics**				
Age, year sold	69.91 ± 4.86	72.61 ± 6.79		0.011*
Gender (Female)	44 (67.69%)	29 (45.31%)		0.013*
Educational level				
≤ *12 years*	9 (13.85%)	17 (26.56%)		0.204
*between 13 and 17 years*	32 (49.23%)	26 (40.62%)		
≥ *17 years*	24 (36.92%)	21 (32.81%)		
Delay between SCD-T and reference CN-Battery, months	-0.44 ± 0.80	1.12 ± 1.36		< 0.001*
**Medical history**				
cardiovascular diseases^¥^	5 (7.69%)	10 (15.62%)	OR: 0.72 ± 0.47	0.615
hypertension^¥^	15 (23.08%)	25 (39.06%)	OR: 0.51 ± 0.22	0.161
diabetes^¥^	2 (3.08%)	8 (12.50%)	OR: 0.38 ± 0.32	0.262
depression^¥^	5 (7.69%)	17 (26.56%)	OR: 0.16 ± 0.09	0.001*
**Reference CNB**				
HAD	8.65 ± 4.50	12.14 ± 6.60	MD: -4.57 ± 1.08	< 0.001*
MMSE^#^	28.95 ± 0.89	24.94 ± 2.40	MD: 1.74 ± 0.14	< 0.001*
FAB^#^	17.31 ± 0.97	14.38 ± 2.43	MD: 1.79 ± 0.18	< 0.001*
FCSRT free recall^#^	34.72 ± 5.19	10.39 ± 7.97	MD: 2.23 ± 0.06	< 0.001*
FCSRT total recall^#^	47.02 ± 1.29	25.25 ± 14.07	MD: 3.94 ± 0.14	< 0.001*
TMT A time, seconds		61.08 ± 42.88		
TMT B time, seconds		145.71 ± 80.16		
TMT B-A, time, seconds		98.24 ± 73.06		
**SCD-T**				
Time to complete SCD-T (*minutes*)	21.67 ± 4.77	40.74 ± 12.06	MD: -18.35 ± 1.67	< 0.001*
MacNair	12.42 ± 6.53	17.78 ± 7.26	MD: -6.81 ± 1.26	< 0.001*
GDS	2.05 ± 1.99	3.17 ± 3.19	MD: -1.48 ± 0.49	0.003*
NCT good answers	32.88 ± 7.55	14.75 ± 8.71	MD: 16.86 ± 1.47	< 0.001*
NCT wrong answers	1.65 ± 1.89	3.48 ± 4.62	MD: -1.08 ± 0.63	0.186
NCT total answers	34.52 ± 6.79	18.23 ± 8.46	MD: 15.78 ± 1.36	< 0.001*
5-WT total score^#^	9.74 ± 0.54	6.31 ± 2.34	MD: 3.11 ± 0.27	< 0.001*
5-WT weighted total score^#^	18.88 ± 1.55	10.88 ± 3.99	MD: 2.63 ± 0.14	< 0.001*
TMT A time, seconds	26.62 ± 13.13	45.61 ± 34.33	MD: -17.63 ± 4.92	< 0.001*
TMT B time, seconds	52.37 ± 23.14	125.42 ± 87.38	MD: -74.48 ± 12.01	< 0.001*
TMT B-A, time, seconds	25.85 ± 23.21	79.83 ± 76.00	MD: -56.77 ± 10.51	< 0.001*
**Acceptability**				
SUS	92.23 ± 10.75	79.49 ± 18.70	MD: 11.39 ± 2.80	< 0.001*
QCT: successful as person of the same age^1^(≥ Good)^¥^	40 (61.54%)	11 (17.19%)	OR: 8.26 ± 3.84	< 0.001*
QCT: representative memory/attention^2^(≥ fairly representative)^¥^	55 (84.62%)	47 (73.44%)	OR: 1.51 ± 0.73	0.395
QCT: perception^3^(≥ Good)^¥^	49 (75.38%)	31 (48.44%)	OR: 5.23 ± 2.33	< 0.001*
QCT: clear instructions^4^(≥ fairly clear)	64 (98.46%)	60 (93.75%)	ND	ND
QCT: requires no explanation by a third party^5 ¥^	60 (92.31%)	46 (71.88%)	OR: 4.57 ± 2.61	0.005*

Data are presented using mean ± standard deviation (SD) for quantitative measures and using number (percentage relative to group) for categorical measures

*Abbreviations*: *CNB* Comprehensive Neuropsychological Battery, *FAB* Frontal Assessment Battery, *FCSRT* Free and Cued Selective Reminding Test, *5WT* five-word test, *GDS* Geriatric Depression Scale, *GLM* Generalized Linear Model, *HAD* Hospital Anxiety and Depression scale, *MD* Mean difference, *MMSE* Mini-Mental State Examination, *NCT* Number Coding Test, *ND* Not Done, *NDG* neurodegenerative disease group, *OR* Odds ratio, *QCT* Questions on cognitive tests, *SCD-T* Santé-Cerveau digital tool, *SE* standard Error, *SUS* System Usability Scale, *TMT* Trail Making Test

^∫^ Coefficient ± standard error of 'Controls' compared to 'Patients' for linear regressions and Generalized Linear Models (GLM) with binomial link (coefficients transformed on the response scale) [MD]; and odds ratio ± standard error of 'Controls' compared to 'Patients' for logistic regressions [OR]

^‡^ For demographics measures, Welch's t test was used on quantitative measures to compare groups and Fisher's exact test, for categorical measures. For reference NPF-Battery, SCD-T measures and Acceptability measures, group effect was tested via GLM adjusted for age, gender and education level. In the Table, when used GLM with logit link and Binomial distribution for group comparison, # symbols added to the score name; when used logistic regression for group comparison, ¥ symbols added to the score name; when used linear regression no symbol added to the score name

^#^ Group comparison was performed through GLM with logit link and Binomial distribution

^¥^ Group comparison was performed through GLM with logit link and Bernoulli distribution (i.e. logistic regression)

^1^ How do you think you did on these cognitive tests compared to others of your age?

^2^ Do you think tests’ results represent your memory and attention?

^3^ How did you feel during the tests?

^4^ Were the instructions clear? Regression logistic could not be performed because of the small sample size who found the instructions unclear

^5^ Would it have been helpful if someone had explained the tests to you and answered your questions before you took them?

The NDG group had a higher cognitive complaint score (Mac Nair 15 items; mean difference estimate (MDE) ± standard error (SE): -6.8 ± 1.3, *p* < 0.001) and self-rated depression score (GDS 15 items; MDE ± SE: -1.5 ± 0.5, *p* = 0.003).

### Comparison of the performance of NDG and Control groups in SCD-T and in the reference *CNB* (Table 1)

NDG patients had significantly worse cognitive tests scores in the SCD-T and the reference NFT-Battery, except for the NCT wrong answers.

### Association between SCD-T and reference *CNB* scores (Fig. 1)

**Fig. 1:**
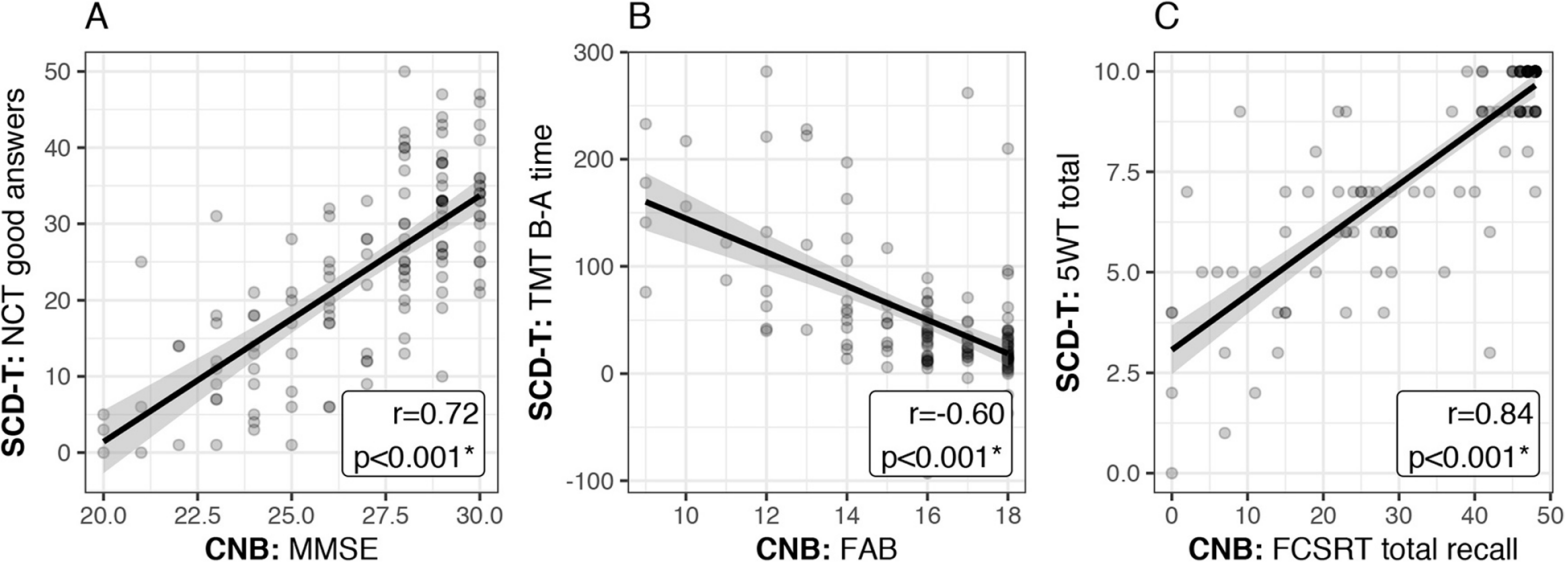
Association between SCD-T scores and those of the reference CNB in the pooled NDG and Controls group. Notes. The scores on the ordinate (preceded by SCD-T) are those from SCD-T and those on the abcissa (preceded by NPF-B) are those from the reference CNB. r is the Pearson correlation coefficient and p the p value from the associated test. Abbreviations: NCT: Number Coding Test; FAB: Frontal Assessment Battery; FCSRT: Free and Cued Selective Reminding Test; 5WT: five-word test; MMSE: Mini-Mental State Examination; CNB: Comprehensive Neuropsychological Battery; SCD-T: Santé-Cerveau digital tool; TMT: Trail Making Test

All the SCD-T cognitive tests scores were significantly associated (*p* < 0.001) with their CNB equivalent when pooling the Controls and NDG individuals. The highest correlation coefficients observed were between the 5-WT and FCSRT total recall (*r* = 0.84, Fig. 1.C) and between the NCT good answers and the MMSE (*r* = 0.72, Fig. 1.A). The SCD-T TMT B-A correlated with the FAB (*r* = -0.60, Fig. 1.B).

### Diagnostic performance

#### Clinician-guided algorithm

Performances of these two-step algorithms are presented in Table 2. The first step of the two-stage ‘sieve approach’ of the clinician-guided algorithm (i.e., the identification of the ASHT using the 5-WT threshold of 9) demonstrated a 92.3% sensitivity and a 80.7% specificity. The second step identified the NCT good answers threshold of [mean ± standard deviation]: 15.0 ± 2.4 as the best discriminant between patients with a 5-WT score of 10 and Controls. As a whole, this clinician-guided algorithm had a 95.4 ± 3.8% sensibility and a 80.5 ± 8.7% specificity.

**Table 2:** Threshold results in combination with the 5WT clinical threshold (9) which maximizes the specificity for a sensitivity of at least 95%

	Optimal threshold	Sensibility	Specificity	Positive predictive value	Negative predictive value
NCT good answers	15.0 ± 2.4	95.4 ± 3.8	80.5 ± 8.7	84.6 ± 6.7	95.1 ± 6.32
NCT total	17.6 ± 3.8	95.3 ± 6.2	76.9 ± 9.7	80.9 ± 5.6	95.3 ± 6.2
NCT wrong answers	4.4 ± 1.9	95.4 ± 3.8	60.1 ± 17.8	71.4 ± 9.3	94.1 ± 4.9
TMT A time	49.4 ± 11.5	95.1 ± 6.6	67.7 ± 11.3	74.8 ± 5.9	94.5 ± 7.2
TMT B time	109.2 ± 13.2	95.4 ± 6.15	72.3 ± 7.84	77.7 ± 4.3	95.1 ± 6.3
TMT B-A time	68.8 ± 23.1	95.1 ± 6.6	70.8 ± 8.9	76.8 ± 4.9	94.7 ± 6.6

Values obtained on the test samples, mean (standard deviation)

*Abbreviations*: *NCT* Number Coding Test, *TMT* Trail Making Test

#### Machine learning classifier

The machine learning classifier considered 8 scores from the 3 SCD-T cognitive tests, the socio-demographic variables, and the medical comorbidities. This classifier obtained a [mean ± standard deviation] 96.8 ± 3.9% sensitivity and a 90.7 ± 5.8% specificity. The positive predictive value (PPV) was 91.6 ± 4.9% and the negative predictive value (NPV), 97.1 ± 3.6%.

### SCD-T acceptability (Table 1)

The System Usability Scale (SUS) was considered excellent in the Control group (mean ± SD: 92.23 ± 10.75) and as good in the NDG group (79.49 ± 18.70) (MDE ± SE: 11.4 ± 2.8, *p* < 0.001). Only 4 patients and 1 control found the instructions unclear. Compared to patients from the NDG group, Control subjects were more likely to think that their performance was normal for age (61.5% vs 17.2%, Odds Ratio (OR) ± SE: 8.3 ± 3.8, *p* < 0.001), to have at least one good feeling during the tests (75.4% vs 48.4%, OR ± SE: 5.2 ± 2.3, *p* < 0.001), to think that an instructor’s explanation would not have been very useful (92.3% vs 71.9%, OR ± SE: 4.6 ± 2.6, *p* = 0.005). Seventy-nine percent of NDG patients and control subjects thought their results were in line with their memory and attention performance; this percentage did not significantly differ between Controls and NDG groups (84.6% vs 73.4%, OR ± SE: 1.5 ± 0.7, *p* = 0.395).

### SCD-T in the post-COVID-19 group

Amongst the 20 post-COVID-19 individuals, 14 had at least 1 test from the reference CNB below a pathological cut-off.

After standardization, all SCD-T and reference CNB cognitive tests scores of the post-COVID-19 group were significantly worse than the Control group (Table 3).

**Table 3:** Comparison between the Controls and post-COVID-19 groups on demographics, reference CNB and SCD-T

	Controls *N* = 65 (82.27%)	Post-COVID-19 *N* = 14 (17.72%)	p ^‡^
**Demographics**			
Age, years old	69.91 ± 4.86	45.07 ± 11.36	< 0.001*
Gender (Female)	44 (67.69%)	11 (78.57%)	0.333
Educational level			
≤ *12 years*	9 (13.85%)	4 (28.5%)	0.082
*between 13 and 17 years*	32 (49.23%)	3 (18.8%)	
≥ *17 years*	24 (36.92%)	7 (50%)	
**Reference NCB**			
MMSE	66.36 ± 4.83	51.78 ± 10.11	< 0.001*
FAB ^•^	1.36 ± 0.97	-0.69 ± 1.86	< 0.001*
FCSRT free recall^+^	0.83 ± 0.92	-0.18 ± 1.71	0.047*
FCSRT total recall ^+^	0.49 ± 0.51	-0.32 ± 1.37	0.0076*
**SCD-T**			
5-WT total score ^°^	-0.52 ± 1.8	-0.68 ± 2.67	0.032*
5-WT weighted total score ^°^	0.23 ± 1.05	-0.95 ± 1.8	0.0082*
TMT A time, seconds ^#^	-1.28 ± 0.75	-0.42 ± 0.77	< 0.001*
TMT B time, seconds ^#^	-1.29 ± 0.44	-0.55 ± 0.92	< 0.001*
TMT B-A, time, seconds^#^	-1.09 ± 0.55	-0.36 ± 0.82	< 0.001*
NCT good answers ^×^	6.32 ± 1.89	3.57 ± 1.69	< 0.001*

Data are presented using mean ± standard deviation (SD) for quantitative measures and using number (percentage relative to group)

*NCT* Number Coding Test; *FAB* Frontal Assessment Battery; *FCSRT* Free and Cued Selective Reminding Test; *5-WT* five-word test; *MMSE* Mini-Mental State Examination; *CNB* Comprehensive Neuropsychological Battery; *SCD-T* Santé-Cerveau digital tool; *TMT* Trail Making Test

^‡^: for categorical and continuous measures, group effect was tested using Mann-Whithney U test

^#^: z-scores computed using GREFEX 2008 norms

^°^: z-scores computed using Croisile et al. 2007 norms

: T-scores computed using Folstein et al. 2002 norms

^+^: z-scores computed using norms provided by neuropsychologists of the IM2A

^•^: z-scores computed using Appollonio et al. 2004 norms

^×^: standard scores computed using WAIS IV

**Fig. 2:**
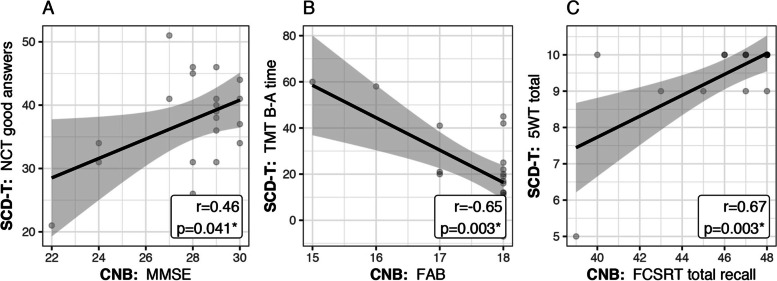
Association between SCD-T scores and those of the reference CNB in the post-COVID-19 subjects. The scores on the ordinate (preceded by SCD-T) are those from Santé-Cerveau digitaltool and those on the abcissa (preceded by NPF-B) are those from the reference NCB. r is the Pearson correlation coefficient and p the pvalue from the associated test. Abbreviations: NCT: Number Coding Test; FAB: Frontal Assessment Battery; FCSRT: Free and Cued Selective Reminding Test; 5WT: five-word test; MMSE: Mini-Mental State Examination; CNB: Comprehensive Neuropsychological Battery; SCD-T: Santé Cerveau digital tool; TMT: Trail Making Test

In the post-COVID-19 group, all the SCD-T tests were significantly correlated (*p* < 0.05) with their equivalent in the CNB. The highest correlation coefficients were observed for the correlation between the 5-WT score and the FCSRT total recall (*r* = 0.67, Fig. 2.C) and the correlation between the TMT B-A and the FAB (*r* = -0.65, Fig. 2.B). The NCT good answers were moderately correlated with the MMSE (*r* = -0.46, Fig. 2.A).

After standardization, the 5-WT total scores and the 5-WT weighted total scores of the NDG participants were significantly worse than those of the 14 post-COVID-19 subjects (Table 4).

**Table 4:** Comparison between the NDG and post-COVID-19 groups on demographics and SCD-T 5-WT

	COVID *N* = 14 (17.95%)	NDG *N* = 64 (82.05%)	p‡
Age, years old	47.34 [39.94, 53.08]	72.81 [66.79, 77.72]	< 0.001*
Gender (Female)	11 (78.57%)	29 (45.31%)	0.037*
Educational level			
≤ *12 years*	4 (28.57%)	17 (26.56%)	0.928
*between 13 and 17 years*	3 (21.43%)	26 (40.62%)	
≥ *17 years*	7 (50.00%)	21 (32.81%)	
5-WT total score°	0.33 [-2.60, 0.60]	-5.87 [-9.67, -2.86]	< 0.001*
5-WT weighted total score°	-1.04 [-2.94, 0.00]	-4.46 [-7.09, -2.66]	0.001*

Data are presented using median [Q1-Q3] for quantitative measures and using number (percentage relative to group)

*Abbreviations*: *5-WT* five-word test

^‡^: for categorical and continuous measures, group effect was tested using Mann-Whithney U test

°: z-scores computed using Croisile et al. 2007 norms

## Discussion

This study demonstrated that all the SCD-T cognitive tests were significantly associated with their equivalent in the clinical setting. Using a straightforward SCD-T setting (the 5-WT with a previously established threshold of 9, followed by a NCT categorization for the individuals performing with a score of 10 on 5-WT) we obtained a 95.4% sensitivity and a 80.5% specificity to discriminate NDG patients from Controls. The diagnostic performance reached a 96.8% sensitivity and a 90.7% specificity using a machine learning classifier based on 8 scores from the 3 SCD-T cognitive tests, socio-demographic variables (age, gender, education level), and medical comorbidities associated with AD (hypertension, diabetes, cardiovascular diseases, and depression). The acceptability of SCD-T was good to excellent according to the severity of the cognitive deficit.

SCD-T was developed to provide a reliable tool to timely identify mild cognitive impairment in primary care. One issue is providing a reliable and easy-to-implement automated tool in the healthcare system that can play a screening role in the general population to optimize the healthcare circuit related to cognitive disorders, as the number of people with cognitive deficits is growing [56]. Still, this screening role must be able to identify mild or subtle cognitive impairments. The three cognitive domains triggered by the SCD-T are coherent with these objectives. The 5-WT is an episodic memory test based on the cueing of the words to be remembered. Therefore, this simple test can isolate the storage deficit characteristic of an amnestic syndrome of the hippocampal type from any other memory disorder, i.e., with a peculiar specificity for AD [31]. In addition, the DSST relies on many executive functions such as central processing, attention, and information processing speed which reflect the overall intellectual general efficiency and is related to the cognitive impairment severity. Finally, the TMT is sensitive to early and subtle cognitive changes, which may occur in AD before the amnestic syndrome. The digital versions of each test were significantly consistent with their reference version, with the highest correlation coefficients (*r* = 0.84) for the 5-WT compared to the FCSRT total recall.

Both diagnostic classifier approaches tested (the clinician-guided one and the machine learning one) provided a good discriminatory capacity for patients of the NDG group with sensitivity higher than 95% and specificity higher than 80%. However, the machine learning algorithm improved by 10% the specificity (80.5 ± 8.7% to 90.7 ± 5.8%).

As our validation study took place during the COVID-19 pandemic, we included subjects with a cognitive complaint following a COVID-19 infection. This group allowed us to test SCD-T in a non-degenerative condition. 70% of the post-COVID-19 individuals tested had at least one deficit in one cognitive domain using the CNB, as reported elsewhere [57, 58]. We replicated a good correlation between the SCD-T and the CNB cognitive tests. Moreover, the performance of patients with NDG diseases was significantly worse than that of post-COVID-19 subjects with cognitive deficit on the reference CNB, demonstrating that SCD-T is able to discriminate different patterns of memory disorders (i.e., amnestic syndrome of the hippocampal type from a memory deficit due to attentional/executive disorder), thanks to the 5-WT.

In the US, screening for cognitive impairment has been encouraged at the Medicare Annual Wellness Visit [2]. To be approved by regulatory agencies for clinical use and covered by health insurance, a cognitive screening tool needs to be robustly validated and impact the clinical diagnosis and therapeutic decisions. Our validation study’s results will help to implement SCD-T as a screening tool in the general population. In France, we plan to propose the following implementation in line with primary care physicians: SCD-T will be available through personal access after a prescription by a clinician. The results and conclusion of SCD-T will be detailed and interpreted based on the algorithms immediately at the end of the test, and available for the prescriber (mainly general practitioners) on a secured web platform with easy access. In case of abnormal results, the general practitioner can refer the subject to the local memory consultation for a more extensive assessment. In case of normal results, the general practitioners will reassure the subject and may also suggest a follow-up evaluation using SCD-T.

Besides the tool’s diagnostic performance, it is also essential that the test is acceptable for people unfamiliar with digital tools. SCD-T was designed so that any individual could complete the test alone (without direct supervision by a healthcare provider). SCD-T had a good to excellent acceptability performance, as illustrated by the high SUS scores in NDG and Control groups. As expected, the test duration was longer in subjects with more severe cognitive impairment.

Numerous digital applications designed to assess cognitive functioning exist; however, the diagnostic performances of most of them have yet to be academically evaluated [11, 59]. Compared to the sensitivities and specificities of 46 digital cognitive tests with self-administered assessment to detect cognitive impairment in elderly participants (mild cognitive impairment and dementia versus controls) [11], the performance of SCD-T was very high, among the best. One of the main limitations of these studies reported in the review is the limited number of subjects in the control groups, with less than 30 participants, in 15 studies [11].

The strengths of our study were the large group size and the detailed diagnostic workup in each group of participants. It is noteworthy that the diagnoses were validated in interdisciplinary meetings based on the neuropsychological, imaging, and CSF data. Hence, SCD-T can detect a mild cognitive impairment but, above all, an amnestic syndrome of the hippocampal type, a core phenotype of typical Alzheimer’s disease [15]. These results are an important added value of our digital application, especially since, to the best of our knowledge, SCD-T has so far the highest diagnostic performances compared to other applications. At least, SCD-T is the only digital application that considers risk factors of cognitive impairment and dementia such as hypertension, diabetes, cardiovascular diseases and depression). By including clinical data and these modifiable risk factors, the machine learning algorithm allowed us to adjust the results provided by the cognitive tests. Moreover, these factors will be mentioned in the Curapy report to the general practitioner to act on these factors and prevent cognitive decline. However, our study had several limitations. The sample size was too small to stratify by age, education, and gender. Then, we adjusted for these three effects in the comparison between NDG and Controls, and age, education and gender were included as features in the machine learning algorithm. For this study, the Controls were selected on the absence of any cognitive impairment on the CNB. They were all cognitively normal. Some of them (34 out 65) had a memory complaint but with a negative CSF AD biomarkers investigation in 32 subjects and 2 were asymptomatic at risk. Our population was highly selected (age between 60 and 85 years, native French speaker ≥ 7 years of schooling, MMSE ≥ 20, population referred to a third care system with AD biomarkers in the CSF…) which is not representative of the subjects consulting their general practitioner for a cognitive complaint, and there was a low number of participants with a non-degenerative cognitive impairment, which is not representative of the subjects assessed in memory consultations [60]. Besides, SCD-T mainly focuses on amnesic and dysexecutive cognitive functions, and our NDG group was mainly composed of typical AD phenotypes. SCD-T will likely be less accurate in screening non-amnestic non-dysexecutive neurodegenerative diseases, such as prodromal Primary Progressive Aphasia or Posterior Cortical Atrophy.

Hence, SCD-T is a simple and fast application with strong diagnostic performance and validated with diagnosis categorization from an expert memory center. This application allows great confidence in identifying an amnestic syndrome of the hippocampal type and, therefore, in detecting AD at a prodromal stage of the disease.

## Conclusion

Healthcare systems need structural and functional innovation toward early detection and diagnosis of cognitive disorders, especially AD. We demonstrated that SCD-T has a high diagnostic performance in identifying prodromal neurodegenerative diseases, especially AD. This opens the opportunity to implement this tool in the general population, to test its ability to guide general practitioners in their referrals to memory clinics and avoid useless referrals of cognitively normal elderly, thus saving costs and improving pathways efficiency. It may also be helpful in pre-screening individuals to be included in AD clinical trials.

## Data Availability

The datasets used and/or analysed during the current study are available from the corresponding author on reasonable request.

## Abbreviations

SCD-T: Santé-Cerveau digital tool
5-WT: 5 Word Test
TMT: Trail Making Test
NCT: Number coding test
AD: Alzheimer’s disease
CSF: Cerebrospinal fluid
COVID-19: Coronavirus disease
SD: Standard deviation
MMSE: Mini Mental State Examination
GDS: Geriatric Depression Scale
DSST: Digit Symbol Substitution Test
WAIS: Wechsler Adult Intelligence Scale
ASHT: Amnestic syndrome of the hippocampal type
CNB: Comprehensive neuropsychological battery, and functional battery
NDG: Neurodegenerative diseases
IM2A: Institut de la Mémoire et de la Maladie d’Alzheimer
FCRST: Free and Cued Selective Reminding Test
FAB: Frontal Assessment Battery
HAD: Hospital Anxiety and Depression
IADL: Instrumental Activities of Daily Living
PASAT: Paced Auditory Serial Addition Test
^18^F-FDG PET-MRI: ^18^F-fluoro-deoxy-glucose positron emission tomography-magnetic resonance imaging
SARS-CoV-2: Severe acute respiratory syndrome coronavirus 2
RT-PCR: Reverse transcription polymerase chain reaction
QTC: Questionnaire on Cognitive Tests
SUS: System Usability Scale
PPV: Positive predictive value
NPV: Negative predictive value
MDE: Mean difference estimate
MDE: Mean difference estimate
SE: Standard error
OR: Odds Ratio
